# Seroprevalence and characteristics of Coronavirus Disease (COVID-19) in workers with non-specific disease symptoms

**DOI:** 10.1186/s12879-022-07461-9

**Published:** 2022-05-20

**Authors:** Wajiha Javed, Syed Hussain Baqar Abidi, Jaffer Bin Baqar

**Affiliations:** Getz Pharma (PVT) Limited, Karachi, Pakistan

**Keywords:** SARS-CoV-2, COVID-19, Seroprevalence, IgM, IgG, Risk Factors, Potential Predictors

## Abstract

**Background:**

The population-based serosurveys are essential for estimating Coronavirus Disease-19 (COVID-19) burden and monitoring the progression of this pandemic. We aimed to assess the seroprevalence of SARS-CoV-2 antibodies and potential predictors of seropositivity in the Pakistani population.

**Methodology:**

This population-based seroprevalence study includes consenting subjects from the workplaces (factories, corporates, restaurants, media houses, schools, banks, and hospitals) located in the urban areas of Karachi, Lahore, Multan, Peshawar, and Quetta. We analyzed each subject's serum sample for SARS-CoV-2-IgM and/or IgG antibodies using UNIPER IgG/IgM Rapid COVID-19 Testing Kit. The subject's demographics, exposure history, and symptoms experienced (in last 7 days) were also obtained. The collected data was analyzed using SPSS version 22.0.

**Results:**

The overall seroprevalence of SARS-CoV-2 antibodies was 16.0% (2810/17,764). The total antibody seropositivity was higher in males than females (OR 1.22, 95% CI 1.110–1.340). The symptomatic subjects had 2.18 times higher odds of IgG seropositivity while 1.2 times for IgM seropositivity than the asymptomatic subjects. The multivariable logistic regression model showed that the odds of SARS-CoV-2 total antibody seroprevalence were affected by the number of dependents (OR = 1.077; 95% CI 1.054–1.099), apparent symptomology (OR = 1.288; 95% CI 1.011–1.643), close unprotected contact with a confirmed or probable case of COVID-19 (OR 2.470; 95% CI 2.164–2.819), traveling history (last 14 days) (OR = 1.537; 95% CI 1.234–1.914) and proximity with someone who traveled (OR = 1.534; 95% CI 1.241–1.896).

**Conclusion:**

We found a reasonable seroprevalence of SARS-CoV-2 antibodies in the studied population. Several factors like the number of dependents, apparent symptoms, close unprotected contact with a confirmed or probable case of COVID-19, traveling history, and proximity with someone who traveled are associated with increased odds of SARS-CoV-2 antibody seropositivity.

## Introduction

After emerging from Wuhan, the novel Coronavirus has rapidly transmitted throughout the world. It is now regarded as a pandemic [1], accompanied by a varying range of mild symptoms including cough, fever, cold and body ache and even severe, like Acute Severe Respiratory Distress (ASRD) become the leading cause of death among these patients [2–4]. According to the World Health Organization (WHO) reports, we now have more than 296 million confirmed COVID-19 cases and more than 5 million deaths worldwide [5]. Locally in Pakistan, the first two cases were reported in February 2020, having a travel history from Iran. Although all preventive measures were taken to contain the disease, unfortunately, around 1,301,141 cases have been identified since then, accounting for 28,961 deaths [6]. But the concern regarding disease control, morbidity, and mortality in Pakistan remains; it is claimed that 415,352 cases have successfully recovered [6], possibly due to low testing rates in Pakistan compared to the rest of the world [7]. One of the significant reasons for limited testing facilities is the restricted healthcare budget and resources [7].

As COVID-19 has become a global threat, early detection and prevention of viral spread have become crucial. Reverse transcription-polymerase chain reaction (RT-PCR) is recognized as the gold standard diagnostic technique as it helps in the early recognition of COVID-19 [8]. Although, due to compromised test sensitivity in association with inadequate sample collection, the time between sample collection, the onset of symptoms, and the fluctuation in viral load [9] linger the duration. However, an easy, sensitive, and inexpensive alternate, i.e., Antibody Test, is now being used to identify SARS-COV-2. Antibody screening through serological assays helps determine the actual frequency and seroprevalence of the virus [10]. The antibodies IgG and IgM can be detected within 1–3 weeks after exposure to Coronavirus. A rapid increase in the antibody level and the seroconversion rate is observed during the first two weeks [11]. Presently the seroconversion rate is being widely studied in order to understand its dynamics, specifically from acute to convalescent phases [12]. It has been found that the IgM and IgA growth after exposure to SARS-CoV-2 is relatively slow compared to other acute viral infections contributing to its heterogeneous pathogenicity [13].

Old age, pre-existing comorbid conditions, low CD3 + CD8 + T-cells levels, high troponin I and d-dimer levels have been recognized as the major risk factors associated with seropositivity and mortality among the infected patients [14–17]. More recently, studies conducted in Shenzhen, including 1286 close contacts (98 COVID positive cases) and Guangzhou including 2098 close contacts (134 COVID positive cases), discovered that significant risk factors for COVID-19 infection were among older age and traveling outside or inside the country, etc [18, 19]. Additionally, a Taiwanese study identified exposure to a confirmed or probable case of COVID-19 as a risk factor [20].

In the current study, we employed a large dataset, including 17,764 participants from Karachi, Lahore, Multan, Peshawar, and Quetta, to estimate the seroprevalence of SARS-CoV-2 antibodies, characteristics, and potential predictors of seropositivity. This serological investigation intends to elaborate Pakistan’s COVID infection statistics under the worldwide pandemic.

## Methodology

### Study design & participants

A population-based study was conducted to investigate the seroprevalence of COVID-19 infection in Pakistan. Sample size was calculated on standard assumptions, which are, 95% confidence interval with 5% margin of error and considering 50% estimated prevalence of COVID-19 during the first wave of pandemic. With these assumptions we had sample size n = 384 for a single center. We aimed to cover approx. 40 centers across Pakistan and achieved a well representative sample size n = 17,764 for final analysis. Participants between 18 to 65 years of age were recruited during April-September 2020 from the workplaces, including factories, corporates, restaurants, media houses, schools, banks, and hospitals, located in Karachi, Lahore, Multan Peshawar, and Quetta through non-probability convenience sampling technique. While non-consenting individuals, PCR-positive at the time of screening, having past 14-days history of COVID-19 and COVID-19 vaccine trials’ participants were excluded.

### Primary & secondary endpoints

The primary endpoint was to assess the seroprevalence of SARS-CoV-2 antibodies (IgM and IgG) among the studied individuals. And the secondary endpoint was to determine the risk factors associated with the seropositivity of these antibodies.

### Procedures & laboratory analysis

For this population-based seroprevalence study, the patient's baseline demographics, medical history, exposure information and details regarding the symptom profile of last 7-days, seropositive rates and the factors associated with SARS-CoV-2 antibodies were investigated at the time of sample collection and noted using a structured questionnaire. The samples were drawn for SARS-CoV-2 rapid antibody testing in a temperature controlled environment by a healthcare professional. IgM and/or IgG antibodies were assessed from human serum using Unite Diagnostic Strip for N Coronavirus (2019-nCoV) IgG/IgM Antibody (Colloidal Gold Immunochromatography Assay) by Uniper Medical Technology Limited, approved by U.S. Food and Drug Administration (US FDA) EUA and certified by European Certified (C.E.).

### Statistical analysis

The data were statistically analyzed using SPSS version 22.0. Categorical variables, including gender, age groups, household size, medical history, symptom profile, contact history, etc., were summarized as frequencies and percentages. Moreover, mean and standard deviation were used for continuous variables like age, weight, and height. The seropositivity rate was calculated by the proportion of test-positive among those tested. Chi-square or Fisher exact test was used to compute the symptomatic association with antibody seropositivity. The Fitting Information Model was used to test the general fit of the model. Moreover, the Multinomial logistic regression was applied to see the association between symptoms and antibodies. A multivariate logistic regression was performed, the IgM and IgG antibody's significant predictors from Univariate analysis were entered into the final model. The forward Wald stepwise approach was used and the significant variables were determined as the independent predictors of seropositivity. A p-value < 0.05 was considered statistically significant.

### Ethical considerations

The study protocol was approved by the Ethical Review Board of AEIRC (Reference no. MU/ECA/20/17820; Dated 2nd April 2020). All the procedures were performed in accordance with the Declaration of Helsinki, and the patient's confidentiality was maintained throughout the investigation. The included patients were well-informed about the study objective and written informed consent was taken from them before inclusion.

## Results

### Participant demographics and exposures

A total of 17,764 individuals were included in the study, with a mean age of 35.68 ± 14.17 years (Range: 18 to 65 years), and of them, 13,003 were males, and 4,761 were females (Table 1).

**Table 1:** Baseline demographic characteristics of the study subjects

Variables	(n = 17,764)
Age; Mean ± S.D. (years)	35.68 ± 14.71
Weight; Mean ± S.D	68.81 ± 15.09
Height; Mean ± S.D	63.01 ± 11.51
Gender	n (%)
Male	13,003 (73.2)
Female	4761 (26.8)
Household size	
< 5	3524 (19.8)
≥ 5	12,542 (70.6)
Not Reported*	1698 (9.6)
Median (IQR)	6 (5–8)
No of dependents	
< 5	9946 (56)
≥ 5	4870 (27.4)
Not Reported﻿*	2948 (16.6)
Median (IQR)	4 (1–5)
Smoker	
No	15,652 (88.1)
Yes	2112 (11.9)
Comorbid conditions	
No	16,678 (93.3)
Yes	872 (4.9)
Not reported﻿*	214 (1.2)
Marital status	
Single	6165 (34.7)
Married	11,599 (65.3)
Monthly income (PKR)	
< 15,000	1433 (8.1)
15,000–30,000	1930 (10.9)
31,000–50,000	1143 (6.4)
51,000–75,000	1100 (6.2)
76,000–150,000	1764 (9.9)
> 150,000	2538 (14.3)
Not Reported﻿*	7856 (44.2)
Recent malaria history	
No	17,381 (97.8)
Yes	164 (0.9)
Not reported﻿*	219 (1.2)
BCG vaccine status	
No	3210 (18.1)
Yes	14,188 (79.9)
Not reported﻿*	366 (2.1)
Close unprotected contact with a confirmed or probable case of COVID-19	
No	14,604 (82.2)
Yes	3024 (17)
Not reported﻿*	136 (0.8)
Travelled outside or inside the country in the last 14 days	
No	16,840 (94.8)
Yes	791 (4.5)
Not reported﻿*	133 (0.7)
Proximity with someone who travelled outside or inside the country in the last 14 days	
No	16,717 (94.1)
Yes	916 (5.2)
Not reported*	131 (0.7)

*Not Reported = Not available (Missing data)

### Prevalence of SARS-CoV-2 antibodies

Out of 17,764 subjects, only 2,810 (16.0%) cases had either or both IgM and IgG positive antibodies. 951 (5.4%) tested positive for IgG antibody, 982 (5.5%) tested positive for IgM antibody, while the remaining had both antibodies (n = 877).

### Symptomatology among study individual

**Fig. 1:**
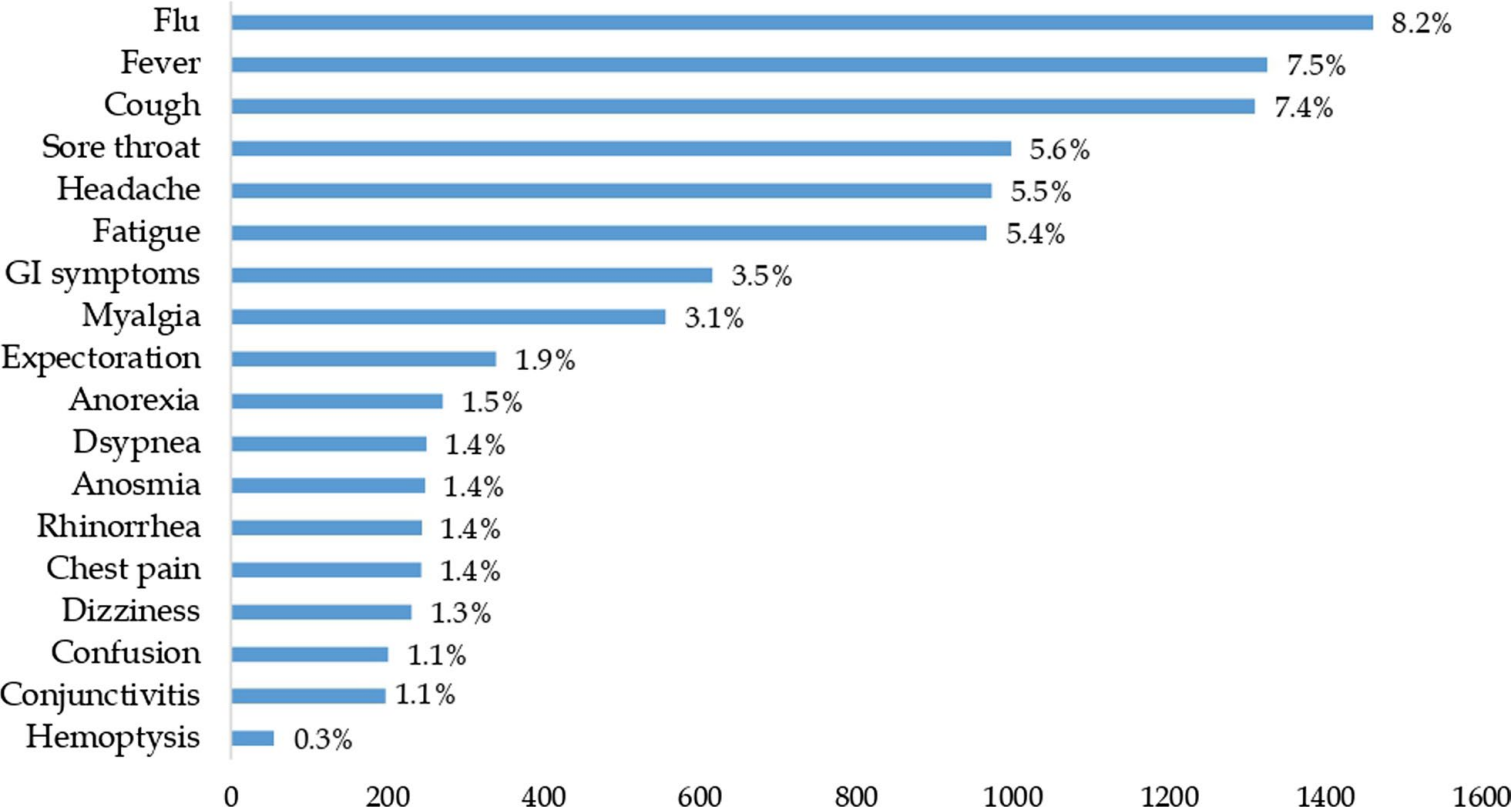
Symptom profile of the study subjects

The data regarding COVID-19 symptomatology for last 7-days was collected before antibody testing. About 8.2% of the study individuals experienced flu-like symptoms, fever (7.5%), and cough (7.4%). While among the less common were sore throat, headache, and fatigue (Fig. 1).

Of the IgM seropositive patients, the most commonly reported symptoms were fever (26.35%), cough (23.39%), and flu (23.02%) as shown in Table 2. Whereas flu (56.29%), cough (48.85%), headache (40.37%) and sore throat (37.14%) were the most frequently reported symptoms among IgG-positive cases.

**Table 2:** Seropositivity by COVID-19 compatible symptoms

Symptoms	IgM seropositivity n (%)	IgG seropositivity n (%)
Flu	428 (23.02)	1029 (56.29)
Fever	490 (26.35)	876 (7.92)
Cough	435 (23.39)	893 (48.85)
Dyspnea	102 (5.48)	145 (7.93)
Anosmia	109 (5.86)	133 (7.27)
Fatigue	343 (18.45)	646 (35.33)
Myalgia	178 (9.57)	403 (22.04)
Anorexia	112 (6.02)	152 (8.31)
Expectoration	94 (5.05)	247 (13.51)
Sore Throat	348 (18.71)	679 (37.14)
Conjunctivitis	99 (5.32)	99 (5.41)
GI Symptoms	160 (8.60)	480 (26.25)
Confusion	86 (4.62)	111 (6.07)
Dizziness	110 (5.91)	131 (7.16)
Headache	266 (14.30)	738 (40.37)
Hemoptysis	23 (1.23)	29 (1.58)
Rhinorrhea	104 (5.59)	147 (8.04)
Chest Pain	117 (6.29)	123 (6.72)
Other Symptoms	–	11 (0.60)

The presence of symptoms showed higher odds of IgG seropositivity (OR = 2.180; CI 1.65–2.88) as compared to that for IgM antibody (OR = 1.190; CI 0.96–1.48) (Table 3).

**Table 3:** Associations between symptoms and seropositivity of SARS-CoV 2 antibodies

Symptomatic	IgM n (%)		OR (95% CI)	p-value	IgG n (%)		OR (95% CI)	**p-value**
	Positive	Negative			Positive	Negative		
No	96 (9)	971 (91)	Ref.	0.107	55 (5.2)	1012 (94.8)	Ref.	0.001*
Yes	1763 (10.6)	14,934 (89.4)	1.190 (0.96–1.48)		1773 (10.6)	14,924 (89.4)	2.180 (1.65–2.88)	

*p-value < 0.05 is considered statistically significant

### Factors associated with anti-SARS-CoV-2 antibodies positivity in the studied population

The odds of having IgM seropositivity were higher among males as compared to females (1.142, 95% CI 1.022–1.276). While the OR = 1.006 (95% CI 0.902–1.122) showed that the chances of IgG seropositivity were similar in the two genders (Table 4). The odds of IgM and IgG seropositivity were similar among all age groups. In association with the SARS-CoV-2 total antibody, the model showed that close unprotected contact with a confirmed or probable case of COVID-19 (OR = 2.437; 95% CI 2.221–2.675), reporting previous symptoms (OR = 1.391; 95% CI 1.152–1.680) and proximity with someone who traveled (OR = 2.270; 95% CI 1.955–2.636) gave increased odds of for positive antibodies.

**Table 4:** Univariate analysis of the factors associated with SARS-CoV-2 Antibodies

Predictors	IgM antibody			IgG antibody			Total antibody		
	OR	95% CI	p-value	OR	95% CI	p-value	OR	95% CI	p-value
Age (years)	1.010	1.006–1.012	0.001*	1.004	1.001–1.007	0.011*	1.003	1.000–1.006	0.024*
Gender (Male)	1.142	1.022–1.276	0.019*	1.006	0.902–1.122	0.914	1.220	1.110–1.340	0.001*
Household size	1.006	0.999–1.013	0.105	1.000	0.993–1.008	0.909	1.001	0.995–1.008	0.735
No of dependents	1.073	1.053–1.093	0.001*	1.065	1.045–1.085	0.001*	1.063	1.047–1.080	0.001*
Smoker	1.129	0.979–1.303	0.096	0.739	0.627–0.871	0.001*	1.069	0.946–1.209	0.283
Married	1.348	1.213–1.498	0.001*	1.101	0.994–1.221	0.066	0.895	0.821–0.976	0.012*
Monthly income (< 15,000)**									
15,000–30,000	1.278	1.003–1.629	0.047*	0.956	0.740–1.235	0.73	1.208	0.980–1.489	0.077
31,000–50,000	1.097	0.828–1.453	0.518	0.867	0.643–1.169	0.35	0.991	0.991–0.776	0.944
51,000–75,000	1.404	1.072–1.838	0.014*	1.246	0.944–1.644	0.121	1.355	1.074–1.711	0.011*
76,000–150,000	1.471	1.155–1.873	0.002*	1.443	1.132–1.841	0.003*	1.514	1.232–1.861	0.001*
> 150,000	1.604	1.280–2.009	0.001*	1.466	1.167–1.842	0.001*	1.484	1.221–1.802	0.001*
Comorbid disease	1.444	1.186–1.757	0.001*	1.244	1.011–1.531	0.039*	1.226	1.029–1.462	0.023*
Recent history of malaria	1.465	0.947–2.265	0.086	0.955	0.569–1.603	0.861	1.253	0.845–1.856	0.262
BCG vaccine	0.928	0.820–1.049	0.231	0.778	0.690–0.877	0.001*	0.907	0.818–1.006	0.064
Symptomatic	1.194	0.963–1.481	0.107	2.186	1.659–2.880	0.001*	1.391	1.152–1.680	0.001*
Close unprotected contact with a confirmed or probable case of COVID-19	2.835	2.550–3.151	0.001*	2.693	2.419–2.997	0.001*	2.437	2.221–2.675	0.001*
Traveled outside or inside the country in the last 14 days	1.871	1.547–2.263	0.001*	1.956	1.618–2.364	0.001*	1.890	1.602–2.230	0.001*
Proximity with someone who traveled	2.597	2.204–3.061	0.001*	2.270	1.913–2.693	0.001*	2.270	1.955–2.636	0.001*

*Significant at p ≤ 0.05 **Reference category

Age, gender, number of dependents, marital status, monthly income, comorbid conditions, close unprotected contact with a confirmed or probable case of COVID-19 travelling history (outside or inside the country), and proximity with someone who traveled (outside or inside the country in the last 14 days), were entered into the final model. In result, number of dependents, marital status, close unprotected contact with a confirmed or probable case of COVID-19 and proximity with someone who traveled were found to be independent significant predictors of IgM seropositivity.

The final model for IgG seropositivity included age, the number of dependents, smoker, monthly income, comorbid disease, BCG vaccine status, close unprotected contact with a confirmed or probable case of COVID-19, travelling history (outside or inside the country in last 14 days), and proximity with someone who travelled (outside or inside the country in last 14 days) (Table 6). No of dependent, smokers, BCG vaccine status, symptomatology, close unprotected contact with a confirmed or probable case of COVID-19, travelling history, and proximity with someone who travelled were the independent significant predictors of IgG seropositivity.

The multivariate regression analysis showed that the potential predictors for total antibody positivity were number of dependents, symptomatology, close unprotected contact with a confirmed or probable case of COVID-19, traveled outside or inside the country in the last 14 days, and proximity with someone who traveled outside or inside the country in the last 14 days (Table 7).

## Discussion

This is the first large-scale serological investigation of 2019-nCoV in Pakistan to the best of our knowledge. As previously published, local studies targeted a specific population or had a small sample size. A serosurvey from Lahore included 154 policemen [21]; similarly, the National Institute of Blood Diseases and Bone Marrow Transplantation from Karachi published the data on SARS-CoV-2 seroprevalence among 380 healthy blood donors [22], and another included 1,675 healthcare workers (HCWs) [23]. Moreover, the preprint data also represent seroprevalence in a limited sample size [24, 25]. The current findings revealed that the overall seroprevalence of anti-SARS-CoV-2 antibodies (IgG and/or IgM) was 16.0%, i.e., 2,842 out of 17,764 inhabitants had developed SARS-CoV-2 antibodies. This figure is significantly higher than that reported in Italy (11.0%), i.e., 6, 30,000 confirmed SARS-CoV-2 cases [26]. This high seroprevalence is consistent with other studies [27–31], 9.7% reported among 2,766 subjects from Switzerland (2020) [27], 13.8% among 674 from Spain (2021) [28], 21.4% of 380 healthy blood donors from Pakistan (2020) [24] and 23% of 390 subjects from Italy (2020) [31].

The seroprevalence of anti-SARS-CoV-2 total antibody was significantly higher among males (OR 1.220, 95% CI 1.110–1.340) (Table 4). In contrast, no significant differences in the seroprevalence of COVID antibodies have been reported among males than females (OR 1.2, 95% CI 0.8–1.8), which is also evidently proven by the existing literature [26, 32, 33]. Furthermore, we found no significant increase in the odds of SARS-CoV-2 antibody seropositivity with respect to age (OR 1.003, 95% CI 1.000–1.006) (Table 4). In contrast, a study reported that the risk of seropositivity was lower among individuals ≤ 20 years of age than those aged > 60 years (OR: 0.72, 95% CI: 0.60–0.87) [34].

In addition to age and gender, close unprotected contact with a confirmed or probable COVID-19 case was also identified as a potential factor affecting the odds of seropositivity (OR 2.437, 95% CI 2.221–2.675) (Table 4) and also confirmed by the forward Wald stepwise logistic regression analysis, including the significant predictors of positive antibody in univariate analysis (Tables 5 and 6). As per the final observed model, number of dependents, symptomatology, closed unprotected contact with the COVID-19 probable or confirmed case, travelling history, and proximity with someone who travelled were the independent significant predictors of seropositivity (Table 7). A study conducted by Rudberg et al. reported that the seropositivity was significantly affected by the patient-related work, i.e., 21% of the 1,764 subjects in patient contact vs. 9% of the 305 subjects without patient contact (OR 2.9, 95% CI 1.9–4.5) [35]. Moreover, the subjects with confirmed COVID patient contact had 1.4 times higher seropositivity odds than those who had non-COVID patient contact (p < 0.05) [35].

**Table 5:** Multivariate Hierarchical Models for the association of predictors with IgM Seropositivity

Final model	AOR	95% CI	p-value
No of dependents	1.075	1.050–1.101	0.001*
Married	1.227	1.030–1.460	0.022*
Close unprotected contact with a confirmed or probable case of COVID-19	2.387	2.060–2.765	0.001*
Proximity with someone who traveled outside or inside the country in the last 14 days	1.895	1.531–2.346	0.001*

*AOR* Adjusted Odd Ratio

*p-value < 0.05 is considered statistically significant

**Table 6:** Multivariate Hierarchical Models for the association of predictors with IgG Seropositivity

Final model	AOR	95% CI	p-value
No of dependent	1.086	1.059–1.114	0.001*
Smoker	0.550	0.424–0.712	0.001*
BCG vaccine status	0.739	0.619–0.882	0.001*
Symptomatic	2.792	1.835–4.249	0.001*
Close unprotected contact with a confirmed or probable case of COVID-19	2.762	2.362–3.230	0.001*
Traveled outside or inside the country in the last 14 days	1.717	1.338–2.203	0.001*
Proximity with someone who traveled outside or inside the country in the last 14 days	1.396	1.092–1.783	0.008*

*AOR* Adjusted Odd Ratio

*p-value < 0.05 is considered statistically significant

**Table 7:** Multivariate Hierarchical Models for the association of predictors with total antibodies

Final model	AOR	95% CI	p-value
No of dependents	1.077	1.054–1.099	0.001*
Symptomatic	1.288	1.011–1.643	0.041*
Close unprotected contact with a confirmed or probable case of COVID-19	2.470	2.164–2.819	0.001*
Traveled outside or inside the country in the last 14 days	1.537	1.234–1.914	0.001*
Proximity with someone who traveled outside or inside the country in the last 14 days	1.534	1.241–1.896	0.001*

*AOR* Adjusted Odd Ratio

*p-value < 0.05 is considered statistically significant.

As for clinical symptoms, we found that symptomatic participants had 2.18 times higher odds of IgG seropositivity and 1.2 times higher IgM seropositivity than the asymptomatic participants (Table 3). The presence of symptoms was significantly associated with seropositivity of IgG antibody and insignificant for the IgM antibody. Interestingly, 9% of the asymptomatic subjects showed IgM antibodies, and 5.2% had IgG antibodies. Also supported by multiple other studies [24, 26], Vena et al. reported seropositivity among 8.6% of the asymptomatic subjects, and a similar local study reported that 53.2% of COVID positive HCWs were completely asymptomatic [26]. These findings indicated that non-apparent infections are common among healthy, active individuals. However, the seroprevalence of SARS-CoV-2 antibodies was reportedly high among symptomatic individuals (p < 0.05). The most commonly reported symptoms of the IgM seropositive patients were fever, cough and flu. Whereas headache, flu, cough and sore throat were the most frequently reported symptoms among IgG-positive cases (Table 2). In contrast, a study described the strongest association of symptoms like anosmia, ageusia, and fever with seropositivity of SARS-CoV-2 antibody [35].

The present study's findings have several implications for pandemic management since a large proportion of COVID infected subjects remain asymptomatic, also supported by the current study findings. Hence, the actual numbers of confirmed SARS-CoV-2 cases might be higher than those reported. Thus, we need to employ stringent screening strategies targeting the infectious spread beyond the current symptom-driven approach [36]. Furthermore, the study limitations must also be discussed; we analyzed the serum samples of only those who voluntarily decided to be tested (workers from selective organizations), suggesting selection bias. Moreover, the prevalence estimates might have varied due to false serology test results and among elderly or immunosuppressed subjects. Hence, the data cannot be generalized to whole population and the objective was to determine the significant characteristics which are still difficult to predict due to varying and non-specific nature of Coronavirus disease.

## Conclusion

In conclusion, the overall SARS-CoV-2 antibodies (IgG and/or IgM) seroprevalence was 16.0%. The multivariate model predicted that number of dependents, symptomatology, close unprotected contact with a confirmed or probable case of COVID-19, travelling history and proximity with someone who traveled are the significant independent predictors of total antibody seropositivity in the studied population. More efforts concerning public health investigations for COVID-19 on a regional and national level should be made to identify the particular risks and outcomes related to SARS-CoV-2 seroprevalence.

## Data Availability

The datasets generated and/or analyzed during the current study are available in the Mendeley Data repository, https://data.mendeley.com/datasets/nyd3vj48kf/1.

## Abbreviations

COVID-19: Coronavirus Disease 2019
SARS-CoV-2: Severe acute respiratory syndrome coronavirus 2
ASRD: Acute Severe Respiratory Distress
WHO: World Health Organization
RT-PCR: Reverse transcription-polymerase chain reaction
US FDA: U.S. Food and Drug Administration
CE: European Certified
EUA: Emergency Use Authorization
SPSS: Statistical Package for the Social Sciences
GI: Gastrointestinal
